# Efficacy of interferon alpha for the treatment of hospitalized patients with COVID-19: A meta-analysis

**DOI:** 10.3389/fimmu.2023.1069894

**Published:** 2023-01-26

**Authors:** Mykhailo Buchynskyi, Iryna Kamyshna, Katerina Lyubomirskaya, Olena Moshynets, Nazarii Kobyliak, Valentyn Oksenych, Aleksandr Kamyshnyi

**Affiliations:** ^1^ Department of Microbiology, Virology, and Immunology, I. Horbachevsky Ternopil National Medical University, Ternopil, Ukraine; ^2^ Department of Medical Rehabilitation, I. Horbachevsky Ternopil National Medical University, Ternopil, Ukraine; ^3^ Department of Obstetrics and Gynecology, Zaporizhzhia State Medical University, Zaporizhzhia, Ukraine; ^4^ Biofilm Study Group, Department of Cell Regulatory Mechanisms, Institute of Molecular Biology and Genetics, National Academy of Sciences of Ukraine, Kiev, Ukraine; ^5^ Endocrinology Department, Bogomolets National Medical University, Kyiv, Ukraine; ^6^ Medical Laboratory CSD, Kyiv, Ukraine; ^7^ Institute of Clinical Medicine, University of Oslo, Oslo, Norway

**Keywords:** COVID-19, interferon-α, IFN-α, mortality, SARS-CoV-2

## Abstract

**Introduction:**

IFN-α intervention may block SARS-CoV-2 replication and normalize the deregulated innate immunity of COVID-19.

**Aim:**

This meta-analysis aimed to investigate the efficacy of interferon IFN-α–containing regimens when treating patients with moderate-to-severe COVID-19.

**Material and methods:**

PubMed, SCOPUS, and ClinicalTrials.gov were searched from inception to 15 January 2022. A systematic literature search was conducted by applying relevant terms for ‘COVID-19’ and ‘interferon-α’. The primary outcome enclosed the all-cause hospital mortality. The secondary outcomes constituted the length of hospital stay; hospital discharge; nucleic acid negative conversion.

**Results:**

Eleven studies are enclosed in the meta-analysis. No significant difference in the all-cause mortality rate was found between the study and control groups (OR 0.2; 95% CI 0.05-1.2; I^2^ = 96%). The implementation of interferon did not influence such outcomes as the length of hospital stay (OR 0.9; 95% CІ, 0.3-2.6; I^2 =^ 91%), nucleic acid negative conversion (OR 0.8; 95% CI, 0.04-17.2; I^2 =^ 94%). Nevertheless, IFN-α treatment resulted in a higher number of patients discharged from the hospital (OR 26.6; 95% CІ, 2.7-254.3; I^2^ = 95%).

**Conclusions:**

Thus, IFN-α does not benefit the survival of hospitalized COVID-19 patients but may increase the number of patients discharged from the hospital.

**Systematic review registration:**

www.crd.york.ac.uk/prospero, identifier (CRD42022374589).

## 1 Introduction

According to the World Health Organization (WHO), the COVID-19 pandemic has affected about 536 million people, resulting in more than six million deaths (1). Even though almost 80% of people infected with COVID-19 have had mild to moderate diseases, an important task is to treat and prevent developing severe and critical conditions (2, 3). Although currently, there are several effective vaccines and targeted antiviral drugs against SARS-COV-2 in the world, such as Molnupiravir and Paxlovid (4), interferons (IFN) have repeatedly attracted the attention of virologists.

Interferons are the most conservative and evolved system of combating viral infection, which is more than 400 million years old (5). Due to their classification, interferons are typically divided into type I, II, and III IFNs. In turn, type I IFNs include IFN-α, IFN-β, IFN-ω, IFN-κ, and IFN-τ (6). Type I IFNs manifest both autocrine and paracrine. These interferons trigger the JAK/STAT pathway to activate diverse genes called interferon-stimulated genes (ISGs). These ISGs work together to stop the viral life cycle at different stages (7). Type I IFNs reduce SARS-CoV-2 replication in Vero E6 cells *in vitro* assays, viral antigen expression, viral load reduction, and plaque reduction (8). In addition, recombinant human interferon-α1b suppresses SARS-CoV-2 more effectively than IFN-α2b *in vitro* (9). IFN-α can be in combination with drugs influencing viral RNA transcription, protein synthesis, and processing to create synergistic consolidations that may be good targets for future preclinical and clinical development to resist emerging and re-emerging viral infections (10).

Many viruses, including SARS-CoV-2, have developed various mechanisms to escape the antiviral function of IFN-I (11). About 10 SARS-CoV-2 proteins were identified as antivirals against IFN (12, 13). SARS-CoV-2 may avert IFN production through mechanisms such as getting away from recognition by pattern recognition receptors, interfering with retinoic acid-inducible gene I or toll-like receptor signaling, and inhibiting phosphorylation of interferon regulatory factor 3 and its activation. Non-structural protein 16 (NSP16) suppresses mRNA splicing, NSP1 leads to total inhibition of mRNA translation by binding to 18S ribosomal RNA at the mRNA entry channel, and NSP8 and NSP9 disrupt protein trafficking across the membrane (14). All these mechanisms lead to a decrease in the production of type I IFN by the affected cell. Even the IFN, which, despite these obstacles, is synthesized and leaves the cell, cannot bind to its receptors. The ORF3a protein blocks the signal that finally enters the target cell and is blocked at the level of formation of transcription factors IRF3, IRF7 or STAT1 (15).

Some other studies have also shown that low levels of type I and III IFNs are partly responsible for the peculiar and inappropriate inflammatory response observed in patients with COVID-19 (16). Different studies have detected solid grounds which advocate the use of type I IFN in combination with other antivirals to achieve positive results in COVID-19 patients. Still, many of them are limited by the number of patients receiving interferon (17). Separate clinical trials have found different outcomes for IFN treatment in COVID-19 (18). There are several meta-analyses on the efficacy of IFN-β (19, 20), but there are no such data on IFN-α that has a wide range of antiviral properties and is used as a first-line drug in the treatment of various viral diseases. Since we need more information about possible treatments for Covid-19, and there is not enough data on the potential of IFN -α against Covid-19, this meta-analysis allows us to understand the effectiveness of this line of therapy.

This meta-analysis aims to evaluate the effects of IFN-α treatment in moderate to severe COVID-19 cases concerning mortality, duration of hospital stay, and a range of other results to help guide the decision to choose this drug as a therapeutic option for this disease treatment.

## 2 Materials and methods

### 2.1 Literature search strategy

**Figure 1:**
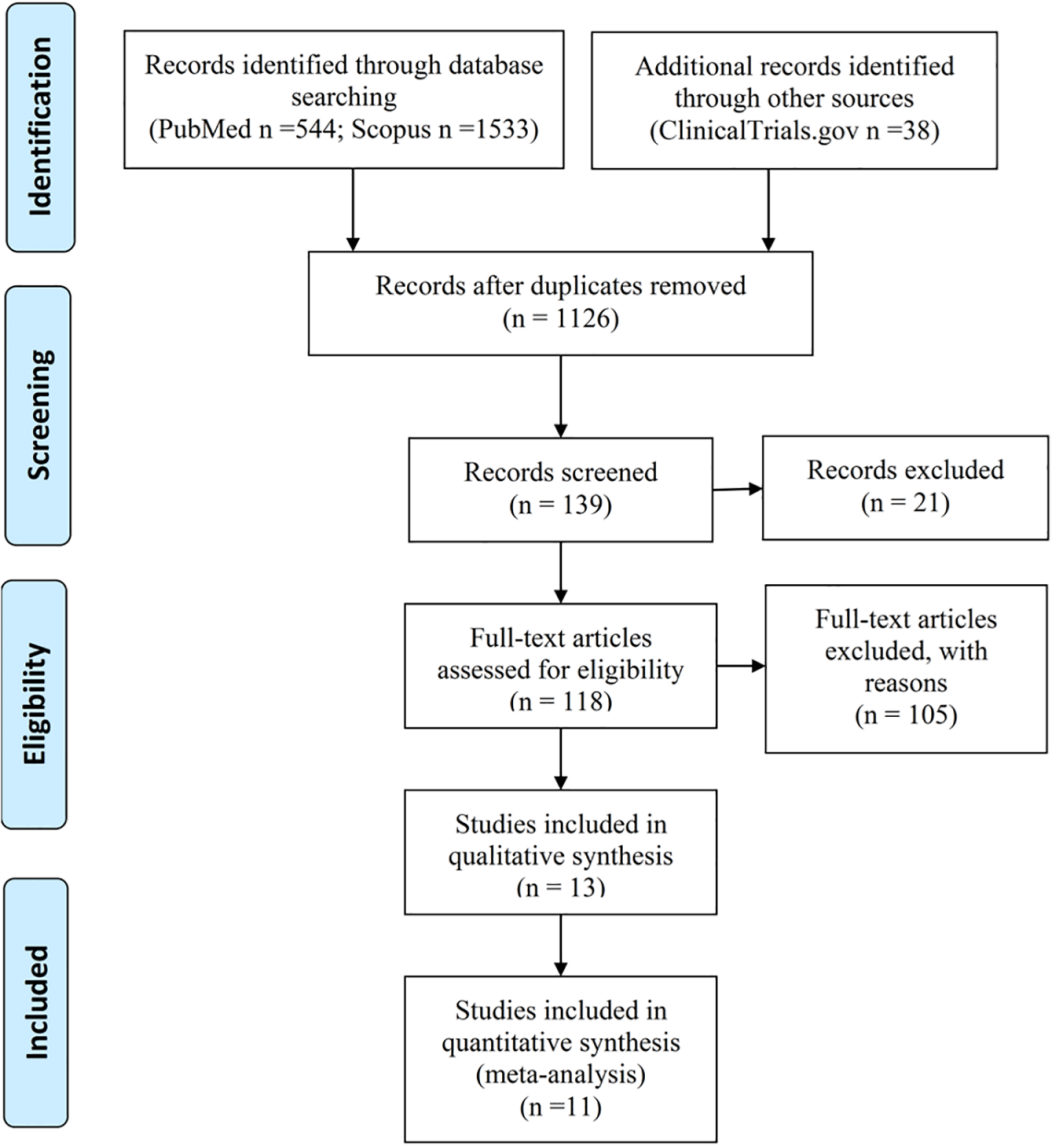
PRISMA flow diagram.

This study followed the Preferred Reporting Items for Systematic Reviews and Meta-Analyses (PRISMA) guidelines (21). A literature search was conducted in PubMed, SCOPUS, and ClinicalTrials.gov for relevant articles from inception to January 15, 2022 (**Figure 1**). For searching in the bibliographic databases we created a research question and three concepts that connected together. Research question: How does interferon alpha affect the treatment of patients with Covid-19. Concept 1: Covid-19: “COVID-19”[Mesh] OR “coronavirus disease*” OR “coronavirus disease 19*” OR “SARS-CoV-2”. Concept 2: interferon alpha: “interferon-alpha*”[Mesh] OR “ IFN -α*” OR “Infa*”. Concept 3: treatment: “treatment*”[Mesh] OR “therapy*”. The literature search was conducted regardless of language or type of publication.

### 2.2 Inclusion/exclusion criteria

Studies that met the following criteria were included (1): examined patients with COVID-19; (2) used IFN-α-containing regimens, as a single agent or in combination, to treat or prevent coronavirus infections; (3) used various treatment options, the standard of care, or placebo as comparators; (4) human studies, despite randomized controlled trials (RCTs), case-control studies, observational studies, cohort studies, or case series; (5) compared the treatment results of IFN with other agents (supportive treatment only, corticosteroids, or between IFNs); (6) reported clinical effectiveness and risk of adverse events (AEs) as study results. The following exclusion criteria were considered: (1) *in vitro* studies or animal models; (2) pharmacological, cellular, molecular, histological, or pathological studies or hypotheses; (3) studies without sufficient data to analyze results; (4) other antiviral therapies that do not involve IFN-α; (5) reviews, commentaries or letters, conference proceedings or abstracts, staged trials or studies without comparative information were ruled out.

### 2.3 Data extraction and quality assessment

Iryna K. and Katerina L. separately screened and reviewed each study. In case of disagreement appeared it was addressed to Nazarii K. After that, they were given access to the full text of the initially selected studies, and the data were entered into Microsoft Excel. For each included study, we considered the following data: year of publication, study design, subjects treated with IFN-α (no treatment), the severity of COVID-19, type of IFN-α, route of administration, and clinical outcomes for IFN-α effectiveness. Each study’s reliable quality and credibility were independently assessed using the Cochrane Assessment of Risk of Bias Version 2 (RoB 2). It evaluated randomization, allocation concealment, blinding, comprehensiveness, and other sources of bias. Response options for each risk of bias judgment were: low risk, some concern, and high risk of bias.

### 2.4 Outcome indicators

Relevant studies included hospitalized patients with moderate to severe COVID-19. The primary outcome was all-cause hospital mortality (quantity of survivors and non-survivors among those who were and were not administered IFN-α). Secondary outcomes involved duration of hospitalization (amount of days the patient spent in the hospital from the day of admission to the day of discharge); discharge from the hospital (quantity of patients discharged from the hospital with no clinical and radiological symptoms and undetected virus by real-time polymerase chain reaction); negative nucleic acid conversion.

### 2.5 Statistical analysis

Data were input in Microsoft Excel. Meta-analysis was performed using the software “Comprehensive meta-analysis” version 3.3.070 using a random and fixed effects model. The respective summary measures of efficacy were evaluated by odds ratio (OR) and/or mean difference (MD) for the appropriate variables, together with the respective 95% confidence interval (CI). The Cochran Q statistic was used for statistical heterogeneity, which was appraised using the I^2^ statistic. I^2^ value lower than 30 was considered “low”, 30-59 “moderate”, 60-74 “significant”, and ≥ 75 “significant heterogeneity”, relatively. The fixed effects model was applied in case the data were homogeneous, and the random effects model was used when the data were heterogeneous. A p<0.05 value was considered statistically significant. The pooled odds ratio (OR) and 95% confidence interval (CI) were calculated to analyze the results.

## 3 Results

### 3.1 Study characteristics

The initial search identified 2115 potential studies (**Figure 1**). After checking the search results and removing duplicates (1126) and irrelevant studies (987), 139 papers were reviewed, some of which (21) were also rejected. Further full-text review and verification of the relevant criteria of 118 studies led to the exclusion of another 105 papers. Of the 13 articles included in the qualitative synthesis, 11 works were included in the meta-analysis.

Of the 11 papers included in the meta-analysis, seven were retrospective cohort studies, two were case-control studies, one was a prospective cohort study, and one was a clinical trial. The number of patients who received appropriate therapy with interferon alfa and a comparison group with standard treatment or therapy with a drug that served as control is presented in **Table 1**. It should be noted the different severity of the course of COVID-19. However, patients with a severe course prevailed (in some studies, data are missing), and various routes of administration of IFN-α (inhalation prevailed in 7 studies; **Table 1**).

**Table 1:** Characteristics of included studies.

Author, year	Study design	The country of origin of the population	Subjects IFNα treatment (not treatment)	COVID-19 severity	Type of IFNα and route of administration	Outcomes on efficacy IFNα
Chen F. et al., 2020 (22)	case-control	Wuhan, China	119 (562)	severe	IFNα, inhalation	survivors and non-survivors
Gong W. et al., 2021 (23)	retrospective cohort	USA; China;	29(179)	severe	not reported, inhalation	survivors and non-survivors, length of hospital stay, nucleic acid negative conversion
Hao S. et al., 2020 (24)	Retrospective cohort	Hangzhou, China	68(36)	mild, severe, critical	IFNα-2b, inhalation	nucleic acid negative conversion, length of hospital stay
Li H. et al., 2021 (25)	Retrospective cohort	Hubei Province, China	1281(756)	mild, severe,	IFNα-1b; IFNα-2a; IFNα-2b, inhalation	survivors and non-survivors, length of hospital stay
Li L. et al., 2020 (26)	Case-control	Wuhan, China	21(92)	not reported	IFNα-2b, subcutaneous (assumed) injection	survivors and non-survivors
Pandit A. et al., 2021 (27)	Clinical trial	India	20(19)	moderate	Pegylated IFNα-2b, subcutaneous injection	nucleic acid negative conversion
Pereda R. et al., 2020 (28)	Prospective cohort	Cuba	152(23)	mild, moderate (assumed)	IFNα-2b, intramuscular injection	survivors and non-survivors, length of hospital stay and discharged from hospital
Pereda R-2. et al., 2020 (29)	retrospective cohort	Cuba	2165(130)	mild, moderate	IFNα-2b, intramuscular injection	survivors and non-survivors, discharged from hospital
Wang N. et al., 2020 (30)	Retrospective cohort	China	216(204)	moderate, severe, critical	IFNα-2b, inhalation	survivors and non-survivors, length of hospital stay
Wong C. et al., 2021 (31)	Retrospective cohort	Hong Kong, China	310(146)	Mild, moderate, severe, critical	IFNα-2b, IFNb-1b, inhalation	survivors and non-survivors, length of hospital stay
Yu J. et al., 2020 (32)	Retrospective cohort	China	852(549)	not rep	IFNα-2b, inhalation	survivors and non-survivors, length of hospital stay

### 3.2 Risk of bias assessment for included studies

**Figure 2:**
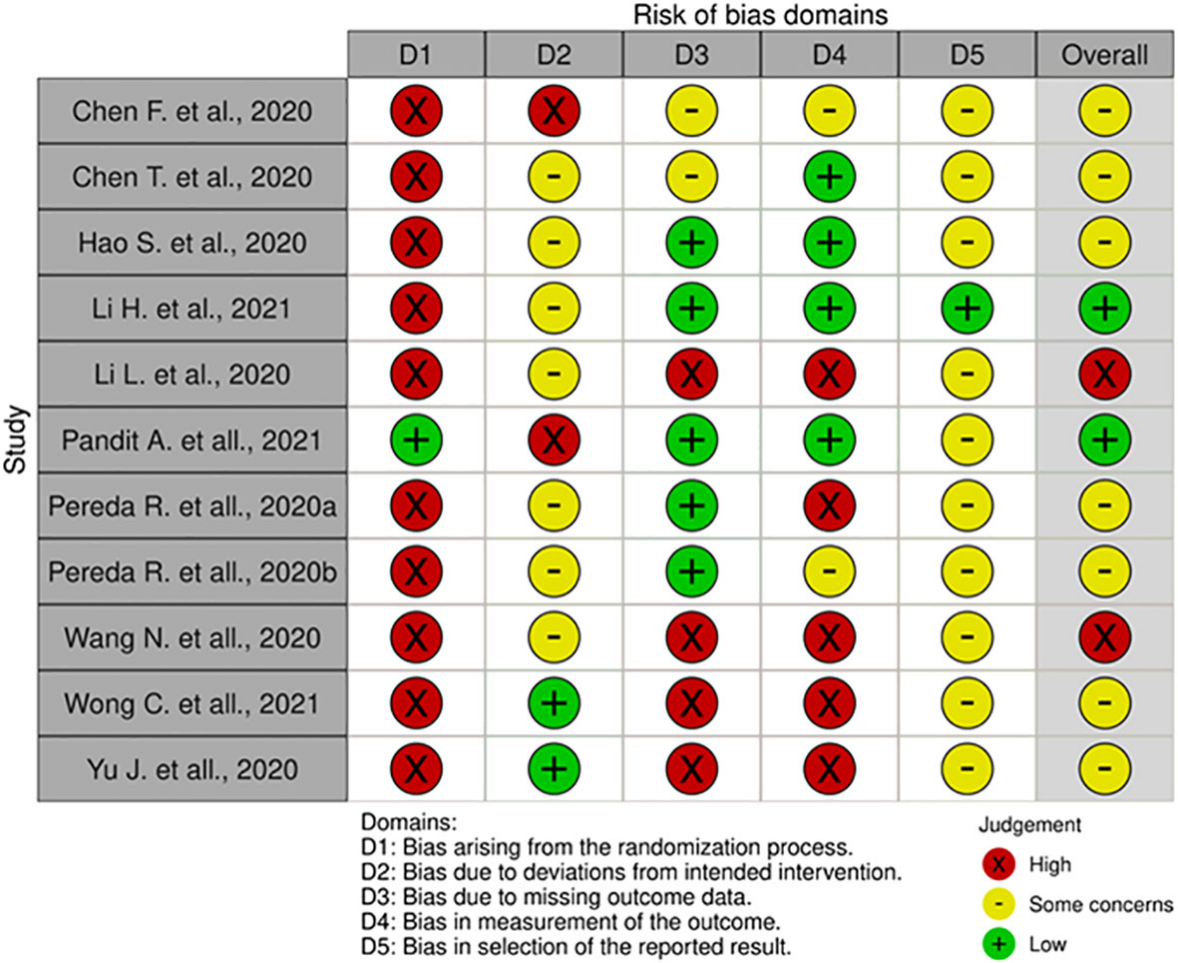
Risk of bias.

Meta-analyses and systematic reviews should consider the included studies’ possible restrictions. The quality of each study was assessed independently by Mykhailo B. and Aleksandr K. using the Cochrane Risk of Bias version 2 (RoB 2) assessment tool, which evaluated randomization, allocation concealment, blinding, completeness, and other sources of bias. Options for assessing the risk of bias were low, high, and doubtful (**Figure 2**). The shortcomings of the works included in the meta-analysis are the following: 1) lack of randomization (all except Pandit A. et al., 2021); 2) no data on blinding of study participants (except for Wong C. et al., 2021, Yu J. et al., 2020); 3) the presence of incomplete data on results and the absence of all possible conclusions in some studies (Yu J. et al., 2020, Wong C. et al., 2021, Wang N. et al., 2020, Li L. et al., 2020). In general, most of the original publications selected for quantitative analysis have a moderate to low risk of bias, except Li L. et al., 2020 (26) and Wang N. et al., 2020 (30) (high risk).

### 3.3 Outcome parameters

#### 3.3.1 Mortality

**Figure 3:**
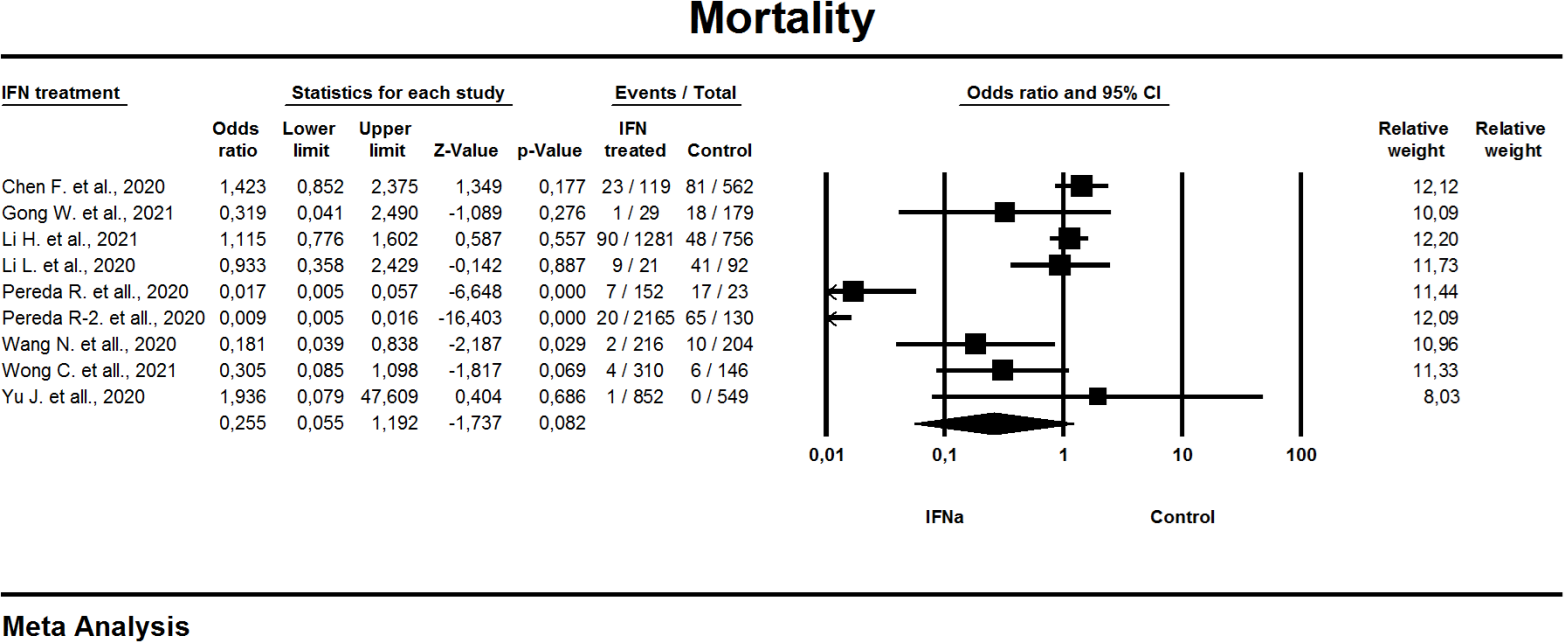
Forest plot mortality (random model).

In nine of the eleven studies included in this meta-analysis, mortality was one of the study outcomes (**Figure 3**). Random effects model was applied to conduct a meta-analysis. The results were entered into the “Comprehensive meta-analysis” program (version 3.3.070), and a calculated OR was used for interpretation. The overall effect estimation [OR 0.2; 95% CI 0.05, 1.2] identifies the non-significant difference between the effect of IFN-α and that of control on mortality (p=0.082). General heterogeneity was high, with an I^2^ value of 96%.

**Figure 4:**
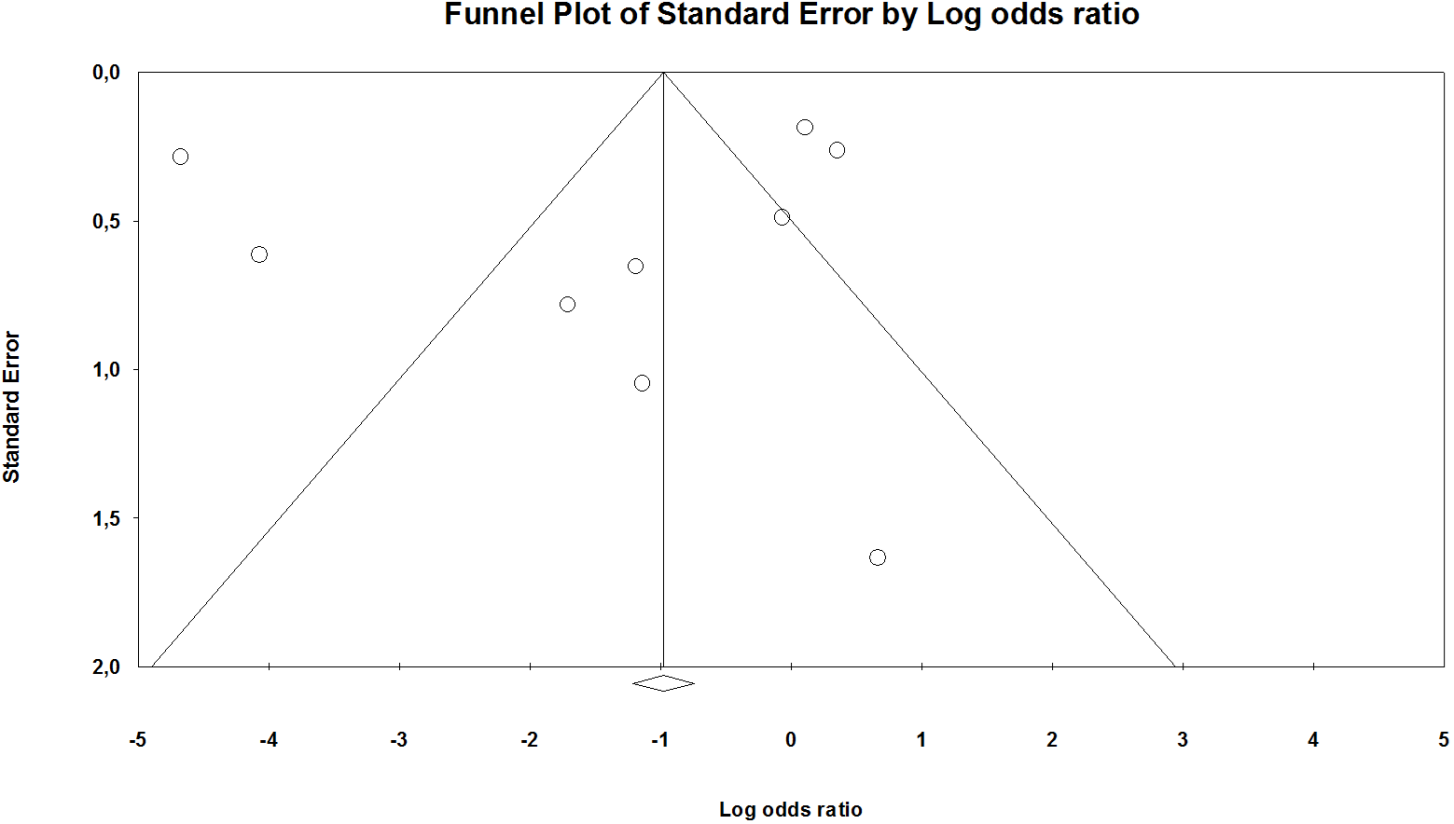
Funnel plot mortality (in **Supplementary Figures**).

The funnel plot test plays a minimal role in cases where the included studies constitute less than 10. Moreover, to fulfill the requirements of the PRISMA checklist, a scatter diagram was generated with the OR on the abscissa and standard error (log [OR]) on the ordinate by applying the inverted funnel plot method. The number of studies included in this meta-analysis was inadequate to specify chance from real asymmetry (**Figure 4**; **Supplementary Figures**). Considering that all other secondary outcomes indices in this analysis were also evaluated for less than 10 papers, we did not conduct the funnel plot and or evaluate the publication bias.

#### 3.3.2 Length of hospital stay

**Figure 5:**
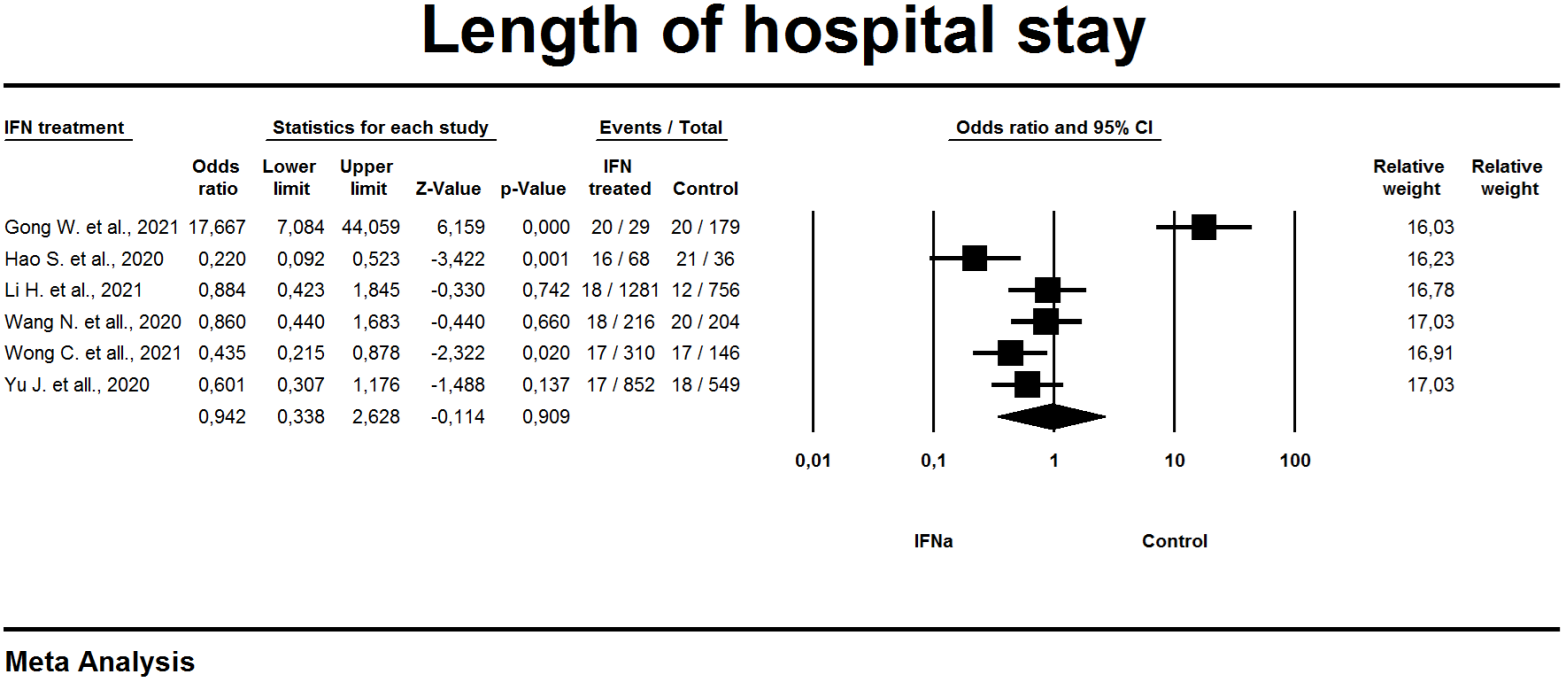
Forest plot length of hospital stay.

Length of hospital stay was one of the study outcomes in six of the eleven studies. A random effects model was used for analysis. Overall effect estimation [OR 0.9; 95% CI 0.3-2.6] did not reveal a significant effect of the prescribed IFN-α on the length of stay in the hospital compared to the control and did not have a statistically significant impact (p=0.91) from the result shown in the control group (**Figure 5**). Significant heterogeneity was found among the studies, with a value of I^2 =^ 91%.

#### 3.3.3 Discharged from the hospital

**Figure 6:**
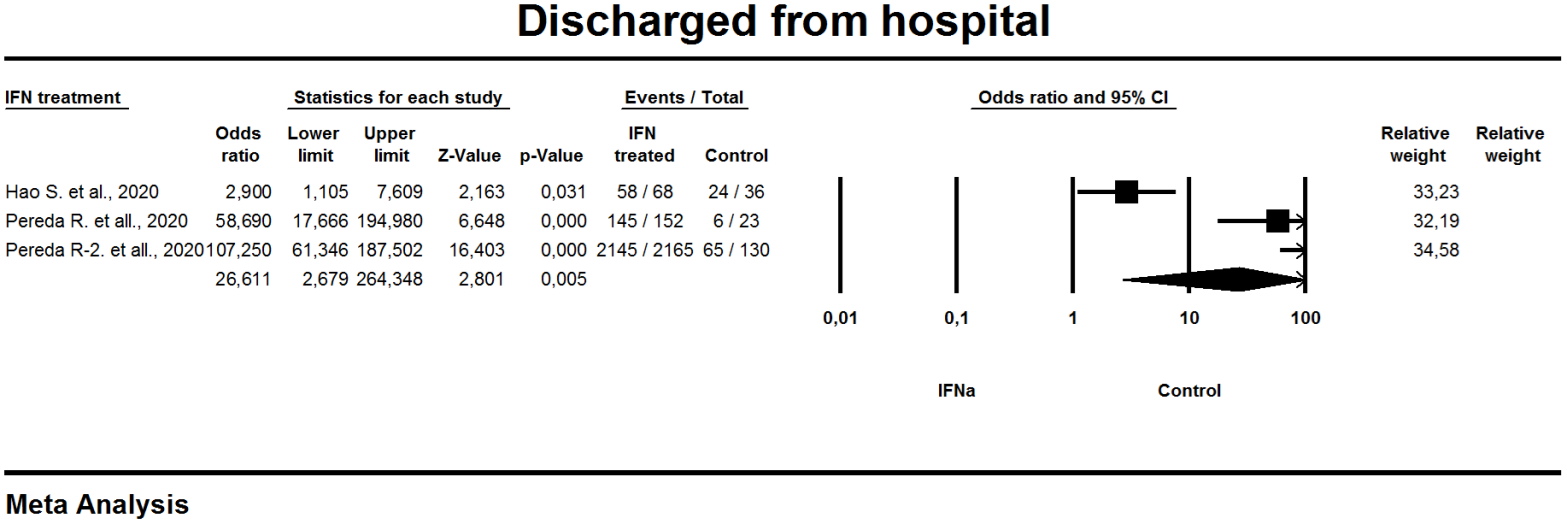
Forest plot discharged from hospital.

Data on patients discharged from the hospital were presented in three studies (**Figure 6**). A random effects model was used in the analysis. Overall effect estimation [OR 26.6; 95% CI 2.6-254.3] indicates the advantage of using IFN-α compared to the control group. P Value = 0.005 excludes the null hypothesis and shows a statistically significant effect of IFN-α use. The detected heterogeneity was high I^2 =^ 95%, correspondently.

#### 3.3.4 Nucleic acid negative conversion

**Figure 7:**
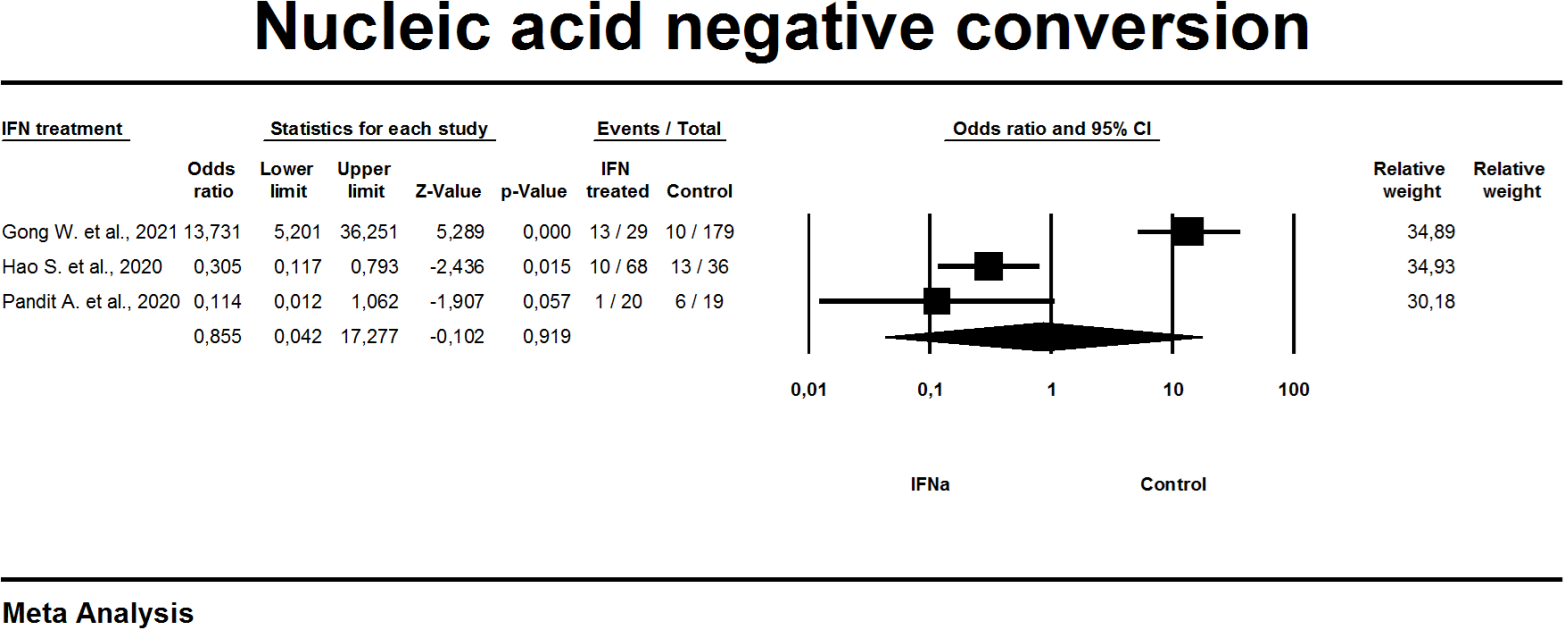
Forest plot nucleic acid negative conversion.

The time of negative seroconversion of the virus was described in three studies (**Figure 7**). Given the presence of significant heterogeneity (I^2 =^ 94%), a random effects model was used in the meta-analysis. Estimating the overall effect [OR 0.8; 95% CI 0.04-17.2] demonstrates the absence of significant changes in this index when prescribing IFN-α treatment compared with the control group (p=0.919).

## 4 Discussion

We found no published meta-analysis evaluating the effectiveness of IFN-α in hospitalized patients with COVID-19. Instead, several meta-analyses assessed the efficacy of the administration of IFN-β. For instance, Kumar S. et al. (2021) (20) conducted a meta-analysis of IFN-β to determine its effect on moderate-to-severe COVID-19 patients. Seven randomized controlled trials (RCTs) were permitted to perform a meta-analysis. The overall effect estimation (OR 0.6; 95% CI 0.9-1.1) determined an insignificant difference between the effect of IFN-β and the control on mortality and length of hospital stay. However, the overall effect estimation (hazard ratio [HR] 1.95; 95% CI 1.36-2.79) was noted for the strong effect of IFN-β on the reduction in time required for clinical improvement in moderate-to-severe COVID-19 patients. In another paper, Chen W. et al. (2022) (19) systematic review and meta-analysis of RCTs were dedicated to investigating the efficacy of IFN-β-containing regimens in treating patients with COVID-19. Eight RCTs were included. A nonsignificant difference in the 28-day all-cause mortality rate was noted between the study and control groups (OR, 0.7; 95% CI, 0.4–1.2; I^2^ = 51%). The study group was admitted to a lower-rate ICU than the control groups (OR 0.6, 95% CI 0.4–0.9; I^2^ = 0%). Subsequently, IFN-β was not connected with an increased risk of any AE or serious AE when compared with the control group. Therefore, IFN-β does not contribute to an increased survival advantage in hospitalized patients with COVID-19 but may assist in reducing the risk of ICU admission. Moreover, IFN-β is a safe agent that can be used in COVID-19 treatment. The most famous study, Solidarity (33), also did not demonstrate the effectiveness of adding IFN-β to therapy. As for the efficacy of IFN-ω, there are currently no meta-analyses and systematic reviews using of this type of interferon in the literature.

The only available systematic reviews of the effectiveness of IFN-α therapy were conducted by Nakhlband et al. (34) and Lu et al. (35). The first group of researchers revealed that the time of viral clearance and polymerase chain reaction negative (days) in most studies were reduced in the IFN-α + standard care group. The mean days of virus clearance in the IFN-α group and the standard group were 27.3 and 32.43. Similarly, the average number of days of hospitalization was also lower in the IFN‐α group (18.55 vs. 24.36). Most of the studies have administered the drug by inhalation.

A comprehensive review of clinical studies in the literature before December 1^st^, 2021 (35), was carried out to find out the current applications of IFN-α. This analysis included facts on the route of administration, the number of patients who received the treatment, the severity at the treatment initiation, age range, the period from the onset of symptoms to treatment, dose, frequency, and duration, as well as safety and efficacy. No evidence was found as opposed to the safe IFN-α treatment for COVID-19. The authors showed that early intervention, either within five days from the initial symptoms or at hospital admission, grants better clinical outcomes, whereas late intervention may cause prolonged hospitalization. Inconsistency in interpretations about IFN‐α responses in patients with coronavirus disease may be attributed to different characteristics in determining moderate, severe, and critical forms of COVID‐19.

### 4.1 Limitations

In most studies, the route of administration of IFN-α treatment included inhalation or nebulization. A number of studies lacked controls. In addition, they were conducted with a small sample size. Some of the studies were retrospective. The adjustment of the included patient groups also left much to be desired. It is beneficial to record the severity of COVID-19 at both hospital admission and treatment initiation, including the worst severity during hospitalization. For reporting IFN-α treatment, it would be handy to include more accurate details, such as dose, frequency, treatment duration; various confounding factors (e.g., age) that could affect outcomes. All 2020 Chinese studies presented in this meta-analysis concern the efficacy of IFN-α against first Strain of 2019-nCoV (C-Tan-nCoV Wuhan Strain).

## 5 Conclusions

Our meta-analysis did not demonstrate the positive effects of IFN-α administration during COVID-19 on such endpoints as mortality, length of hospital stay and nucleic acid negative conversion. The potential explanation could be the low number of eligible studies, different dosing of IFN-α, and the presence of confounding factors, such as concomitant pharmacotherapy. Additionally, due to the heterogeneity of the disease among individuals, probably owing to different factors, such as genetics, age, and gender, the response to anti-COVID-19 treatment may be variable. However, this study prioritizes the use of IFN-α, considering an increase in the number of patients discharged from the hospital.

## Data availability statement

The raw data supporting the conclusions of this article will be made available by the authors, without undue reservation.

## Author contributions

MB and IK contributed to the methodology. KL contributed to the software. OM contributed to validation. MB and NK contributed the formal analysis. NK - data curation; AK – writing of original draft preparation; AK and VO - writing—review and editing; AK - project administration. All authors have read and agreed to the published version of the manuscript. All authors contributed to the article and approved the submitted version.
