# Supplementary figures and images for: Efficacy of interferon alpha for the treatment of hospitalized patients with COVID-19: A meta-analysis
Source: Front Immunol. 2023 Jan 26;14:1069894. doi: 10.3389/fimmu.2023.1069894 (PMC9909279; doi:10.3389/fimmu.2023.1069894)

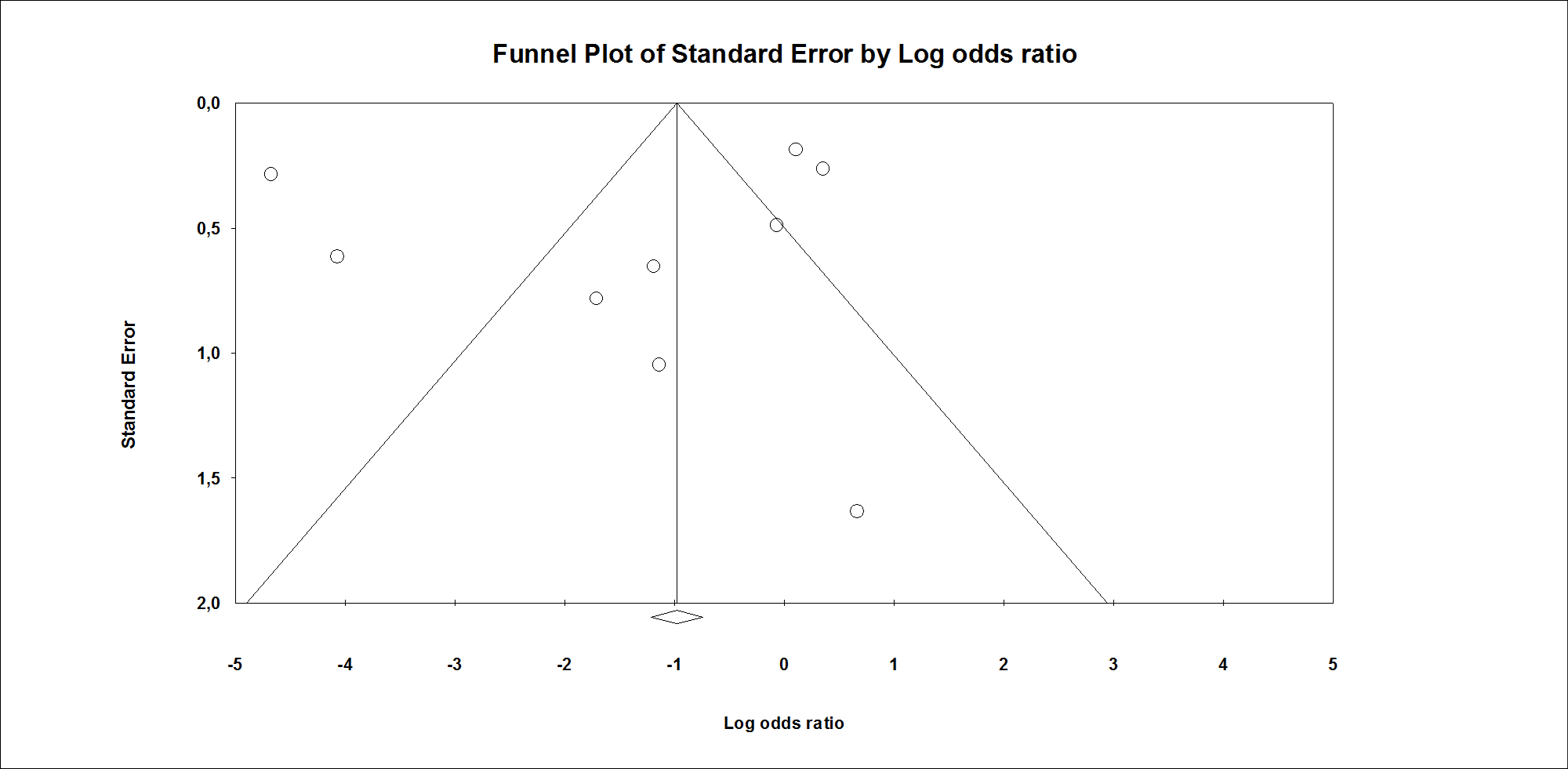

Supplement: Supplementary file 1 [file Image_1.tif]
